# Draft genome sequence of the *Saccharomyces cerevisiae Spy*Cas9 expressing strain IMX2600, a laboratory and platform strain from the CEN.PK lineage for cell-factory research

**DOI:** 10.1128/mra.00550-23

**Published:** 2023-12-22

**Authors:** Marcel van den Broek, Raul A. Ortiz-Merino, Nicole X. Bennis, Anna K. Wronska, Else-Jasmijn Hassing, Pascale Daran-Lapujade, Jean-Marc G. Daran

**Affiliations:** 1 Department of Biotechnology, Delft University of Technology, Van der Maasweg, Delft, the Netherlands; University of California Riverside, Riverside, California, USA

**Keywords:** *Saccharomyces cerevisiae*, CRISPR, biotechnology, cell factory, metabolic engineering

## Abstract

The biobased-economy aims to create a circular biotechnology ecosystem to transition from a fossil fuel-based to a sustainable industry based on biomass. For this, new microbial cell-factories are essential. We present the draft genome of the CEN.PK-derived *Saccharomyces cerevisiae Spy*Cas9 expressing strain (IMX2600), that serve as chassis of new cell-factories.

## ANNOUNCEMENT

Strains belonging to the CEN.PK family were created as part of a multidisciplinary project funded by the Volkswagen Foundation in 1993–1994 (1). This lineage was chosen as a model for physiology and metabolic engineering research. With advent of CRISPR editing technology (2, 3) and increasing complexity of genetic engineering strategies that involve iteration of transformation rounds, chromosomal integration of *Spycas9* can accelerate strain engineering (4). To this end, the *Saccharomyces cerevisiae* strain IMX2600 (MATa *MAL2-8c SUC2 can1*Δ::*Spy*cas9-natNT2) was developed from the prototrophic strain CEN.PK113-7D (MATa *MAL2-8c SUC2*) (5). IMX2600 have been used in constructing microbial cell factories (6–8). To ensure accurate sequencing analysis during construction and/or subsequent adaptive laboratory evolution strategy, a reference genome sequence of this platform strain is essential.

A −80°C stocked glycerol vial of the strain IMX2600 was inoculated and grown in 500 mL shake flasks with 100 mL YPD complex medium (10 g L^−1^ Yeast extract, 20 g L^−1^ BactoPeptone, 20 g L^−1^ glucose). Total DNA was extracted using the YeaStar genomic DNA kit from Zymo Research (Zymo Research, Irvine, CA). Shotgun library preparation using Oxford_Nanopore Technologies' SQK-LSK109 kit (ONT, Oxford, UK) was sequenced on a MinION MK1B device with R10 flow cell. Raw FAST5 signal files were base-called using GPU Guppy 4.0.11 in high-accuracy mode. After filtering for quality and length, 974,343 reads were obtained, yielding 8.69 Gb (N50: 14,169 bp), representing ~724× coverage of a *S. cerevisiae* genome (Table 1). Canu version 2.0 with settings genomeSize = 12 m, useGrid = 0 and nanopore-raw (9) was used for *de novo* assembly yielding full-length chromosomes from telomere to telomere. Nucmer from the MUMmer package (Version 3.1) (10) was used to align and trim the 2µ plasmid and the mitochondrial DNA [nucmer (-maxmatch-nosimplify)]. For error correcting the assembled genome sequence, a 300 bp read length TruSeq PCR-Free Illumina library (Illumina, San Diego, CA) with a 550 bp insert-size yielding 2,793,193 paired reads for a total of 1.6 Gigabases (133-fold coverage) was sequenced on a MiSeq (Illumina). Reads quality was assessed with FastQC v0.11.5. The genome was polished by mapping the untrimmed Illumina reads with Burrows-Wheeler Aligner (BWA version 0.7.15-r1142-dirty; default parameters used) (11, 12) to the assembly and further processed with SAMtools (version 1.3.1) (13, 14) and polished once by Pilon (version 1.18) with settings fix all (15). This yielded a genome assembly of 12.26 Megabase (*N*_50_ = 915,509 bp) composed of 16 chromosomes, a 86,554 bases mitochondrial genome and a 6,318 bases 2µ plasmid (Table 1) with a GC content of 38.26%. CHRXII that harbors the rDNA locus was assembled in one contig of 1,099,799 bp long and four smaller contigs (2*x*~8 kb +1*x* ~55 kb +1*x* ~100 kb) consisting exclusively of rDNA repeats that were not added to the assembly. The annotation of the polished assembly was performed using Funannotate v1.8.15 (https://github.com/nextgenusfs/funannotate) (16). The “predict” (Gene prediction) step was performed using the command (--species "Saccharomyces cerevisiae" --strain IMX2600—augustus_species saccharomyces_cerevisiae_S288C). Interproscan (version 5.25–64, with “-goterms”) was applied in the “predict” step and used as input in the (Functional) “annotate” step. A total of 5,533 coding sequences and 287 tRNA’s were identified.

**TABLE 1:** IMX2600 assembly data and accession numbers at NCBI repositories (https://www.ncbi.nlm.nih.gov/)

Bioproject	PRJNA976676	https://www.ncbi.nlm.nih.gov/bioproject/PRJNA976676
Biosample	SAMN35437089	https://www.ncbi.nlm.nih.gov/biosample/?term=SAMN35437089
	Assembly size (Mb)	12.26
	GC content (%)	38.26
	N50 (bp)	914,509
Experiment	Oxford nanopore	SRX20564515
	Illumina	SRX20564514
SRA	Oxford nanopore	https://trace.ncbi.nlm.nih.gov/Traces/?view=run_browser&acc=SRR24792116&display=metadata
	Illumina	https://trace.ncbi.nlm.nih.gov/Traces/?view=run_browser&acc=SRR24792117&display=metadata
Assembly (annotated)	CP127195– CP127212	https://www.ncbi.nlm.nih.gov/nuccore?term=976676%5BBioProject%5D

## Data Availability

The genome sequencing and assembly of *Saccharomyces cerevisiae* strain IMX2600 have been deposited as Bioproject at NCBI under accession number PRJNA976676 (Table 1).
